# Impact of Carbon Particle Character on the Cement-Based Composite Electrical Resistivity

**DOI:** 10.3390/ma14247505

**Published:** 2021-12-07

**Authors:** Vít Černý, Grigory Yakovlev, Rostislav Drochytka, Šimon Baránek, Lenka Mészárosová, Jindřich Melichar, Radek Hermann

**Affiliations:** 1 Faculty of Civil Engineering, Institute of Technology of Building Materials and Components, Brno University of Technology, Veveří 95, 602 00 Brno, Czech Republic; drochytka.r@fce.vutbr.cz (R.D.); baranek.s@fce.vutbr.cz (Š.B.); meszarosova.l@fce.vutbr.cz (L.M.); melichar.j@fce.vutbr.cz (J.M.); hermann.r@fce.vutbr.cz (R.H.); 2 Department of Geotechnical Engineering and Building Materials, Kalashnikov Izhevsk State Technical University, Studencheskaya Str. 7, 426009 Izhevsk, Russia; gyakov@istu.ru

**Keywords:** cement composite, graphite particles, limestone, resistivity, volume resistivity

## Abstract

Electroconductive cement-based composites are modern materials that are commonly used in many industries such as the construction industry, among others. For example, these materials can be used as sensors for monitoring changes in construction, grounding suspension, and resistance heating materials, etc. The aim of the research presented in this article is to monitor the impact of carbon particle character on cement-based electroconductive composites. Four types of graphite were analyzed. Natural and synthetic types of graphite, with different particle sizes and one with improved electrically conductive properties, were tested. For the analysis of the electrical conductivity of powder raw materials, a new methodology was developed based on the experience of working with these materials. Various types of graphite were tested in pure cement paste (80% cement, 20% graphite) as well as in a composite matrix, which consisted of cement (16.8%), a mixture of silica sand 0–4 mm (56.4%), graphite filler (20.0%) ground limestone (6.7%) and super plasticizers (0.1%). The resistivity and physical-mechanical properties of the composite material were determined. Furthermore, the resistivity of the test samples was measured with a gradual decrease in saturation. It may be concluded that graphite fillers featuring very fine particles and high specific surface are most suitable and most effective for creating electrically conductive silicate composites. The amount, shape and, in particular, the fineness of the graphite filler particles thus creates suitable conditions for the creation of an integrated internal electricity-conductive network. In the case of the use of a coarse type of graphite or purely non-conductive fillers, the presence of an electrolyte, for example, in the form of water, is necessary to achieve a low resistivity. Samples with fine types of graphite fillers achieved stable resistivity values when the sample humidity changed. The addition of graphite fillers caused a large decrease in the strength of the samples.

## 1. Introduction

The cement composite material may be generally characterized as a material of the structure that contains a bonding agent on the basis of cement, a specific type of filler, water, and other components and admixtures [1,2]. The mutual synergic effect of all these components determines the resulting properties of the composite. The wide application options of cement composites are tied to the components used. Among them are various types of concrete, mortars, sealers, and toppings, as well as electrically conductive cement composites [3,4,5].

Electric conductivity is a property indicating the material’s ability to conduct an electric current, indicated by the letter G, and its measuring unit is siemens (S) [6]. Free electrons or ionized atoms or molecules may transmit electric charges in different types of materials [7]. Among the basic types of electric conductivity in conductive materials are direct contact conductivity, tunnel or flashover conductivity or ionic conductivity. The electrons travel through conductive elements of the material (conductive filler), while ions travel through the matrix material where they serve as electrolytes [8,9,10]. There are two electrical conductivity mechanisms of percolative-like behavior, described by (a) electron hopping at the nanoscale and (b) conductive networks formed at the microscale [11,12].

High electrical resistivity of concretes was confirmed by different authors. Electrical resistivity of open-air dried concrete was measured as 6.54 × 10^5^–11 × 10^5^ Ω·cm [9,13,14,15,16,17,18]. In addition, electrical resistivity of saturated and dry concrete were reported as 10^6^ Ω·cm and 10^9^ Ω·cm, respectively [4,13,19]. Electrical resistivity at 28 days of curing, measured with an inter-electrode spacing of 7 cm, was found to be 820, 349 and 78 Ω·cm for mortars reinforced with 1%, 3% and 5% volume fractions of carbon fibers, respectively [20].

Construction materials with electrical conductivity find their use in the removal of snow [21], protecting buildings from static electricity and lightning [22], layout of the grounding system [23,24] in preventing steel structures and strengthening layers of steel in concrete buildings against corroding [25], and electrically conductive composites for protection against electrocorrosion [26,27,28]. Afroughsabet and Ozbakkaloglu [29] pointed out that electrical resistivity has a significant role in the determination of concrete’s durability regarding corrosion [30]. Concrete, which does not lack moisture, presents better performance under lightning conditions, for example, as lightning protection, decreasing the impulse impedance of a grounding rod at lower levels of concrete construction [31]. 

Generally, it could be said that cement composites show slight electron movements due to the presence of n-type conductivity [2]. Among other influences, the volume of the aggregate in the concrete has a reducing effect on the electrical conductivity of concrete. Electrical conductivity of concrete decreases with an increase in the volume of aggregates in [30], due to the dilution effect of conductive ions in pore solution and the adsorption of alkalis on the surface of aggregates. Some conductive ions can be extracted from aggregate into pore solution and increase the electrical conductivity of concrete [32]. The electrical conductivity reflects the percolation and connectivity of the liquid phase in pores as the conductivity of solid phases is negligible [33]. Mortar is a poor electrical conductor, particularly under dry conditions, so its usage is limited in electrical applications [25].

The distribution of the electric current within the sample is affected by a number of external parameters [2]. Otten focuses on monitoring the effects of humidity on electric conductivity, comparing samples treated with hydrophobic impregnations with those untreated and coming to the conclusion that, with increasing material moisture content, the electrical resistivity decreases, while also the temperature of the samples has an effect on electrical resistivity (he monitored 4, 20 and 36 °C) in contrast to what has been reported for cement-CNF composites by Gawel et al., which behave in the opposite way (with decreasing material moisture, the resistivity decreases) [34,35]. The increase in moisture content does not necessarily lead to the same increase in conductivity. There is a point in which moisture increase leads to the dilution of the ionic species and this causes a decrease in ionic strength [36]. Conduction above freezing temperatures depends on the size and connectivity of the capillary pore network, with conductivity generally decreasing with w/c ratio and temperature and increasing with increasing frequency due to the relaxation of polarization processes. Below freezing temperatures, it is shown that electrical conduction is also influenced by volumetric ice content [37].

Concrete is a dielectric material. Since concrete is a highly heterogeneous material, finding an optimal technique in order to understand the behavior of its complex microstructure [38] and to discriminate the electrical properties of diverse components such as the hydrated cement paste, the aggregate skeleton and the interfacial transition zone could be difficult [39]. The electrical resistivity of concrete is affected by various factors, including connectivity of pores, porosity, the conductivity of the pore solution, moisture content, and temperature, etc. [35,40,41]. During the design of the composites, the electronic transport, Seebeck effect, Thompson effect, electron contributions, percolation and molecular dynamics must also be considered [2].

The addition of a conductive admixture may transform a cement-based composite into a conductive material [42]. Conductive concrete is prepared by mixing cementitious materials with conductive components [43]. In order to improve the electrical conductivity, the authors add steel fibers and microfibers [43,44,45,46], steel shavings [43,45], graphite powder [47,48], graphite and carbon fibers (CF) [49,50,51] and microfibers (CMF) [19,44,52,53,54,55,56,57,58], carbon nanofibers (CNF) [59], carbon nanotubes (CNT) [11,52,60,61,62,63,64,65,66], graphene [67,68], anthracite [69] and carbon black powder [70,71] into cementitious composites. Liu [72] focuses on the electric conductivity of soot. Some authors explore the effects of adding ash on electric conductivity [73]. A number of authors also focus on increasing conductivity of asphalt mortars with the use of conductive fillers [74,75,76,77]. 

Azhari and Banthia reported that the addition of CF and CNT at rates of 15.0% and 1.0%, respectively, to cementitious composites significantly increased the electrical conductivity and improved the piezoresistive properties [62]. Kim et al. investigated the influence of CF on the homogeneity of conductive pathways in cementitious composites incorporating CNT and reported that the addition of CF to the composites improved the electrical stability by forming hierarchical conductive networks [63]. Rovnaník studied alkali-activated composite with sodium silicate and cinder, comparing the electric properties of this composite with a standard cement binder [78]. Lunak examined the alkali-activated composite materials based on slag with the addition of different amounts of micronized natural graphite with the dosage of 1–10 wt.% of graphite powder (with 1% increments) [79]. Plugin [26] determined that an increase in the content of graphite filler from 0 to 10–15% in the composition causes a tenfold decrease in its electrical resistivity, and the further increase in graphite content in the mixture does not significantly reduce the electrical resistivity, but an increase in the content of graphite filler (especially with larger particles—60% under 160 µm) causes a decrease in strength [26]. A study published by Nayak focuses on the use of metallic waste iron powder in the cement matrix and replaces the binding agent with it in the composite (up to the amount of 40%) on the basis of a comparison with a mathematical model, monitoring electric conductivity of the composite [80]. Çınar added 0.1, 2 and 3 percent of carbon black to the concrete instead of cement by weight and the concrete was produced by an electrical curing method, and it was observed that the porosity in concrete increased with increasing carbon black ratios [18]. Norambuena-Contreras focuses on electrical resistivity of cement composite with the use of waste steel wool fibers and steel shavings. In order to ensure perfect homogenization and distribution throughout the entire volume of the composite, the particles are mechanically cut and sieved to facilitate the mixing and compaction process of the raw materials [81]. According to Azhari [62], from the perspective of use, suitable conductive fillers have elongated shapes because (provided they are well mixed) they form a “network” inside the composite that better conducts electric current. This is caused by the fact that there is a higher likeliness that some particle of the elongated filler will be closer to another particle of the filler than it would be in the case of round particles that tend to be isolated and thus a higher number of them would be required. He [58] uses conducive aggregate as the filler, produced from graphite and clay, and adds it to mortars along with carbon fibers. Guo produces a conducive aggregate loaded with modified agar solution with graphite powders (with imported NaOH and NaNO_2_, which were added in agar gel to further increase its ionic conductivity) imported into the pore structure of expanded clay aggregates [82]. Braganca concludes that the deposition of Friedel’s salts in the cementitious matrix and the high formation of corrosion products tend to increase the electrical resistivity of the specimens [83]. The reduction of density in cementitious materials directly affects their compressive strength, but the improvement shown can be caused by a graphite flake-shape. Isostatic graphite distribution in the cement mass also can improve the stress transmission as shown in other studies with graphite [84,85]. Graphene (which is fundamentally a single layer of graphite) is characterized by an extremely high conductivity, which is two orders of magnitude greater than that of graphite [86]. As the substitution of PC by FA increases, the electrical resistivity of material at later ages also increases [87]. Limestone powder has been used previously in order to counteract the delays in setting times encountered in most concretes made with FA; however, some works have evaluated the effects of the combination of limestone powder and FA on the shrinkage and durability of concrete [87].

Belli conducted testing of electrical conductivity in order to measure the impedance of the different mortars as a function of the curing time [88]. Pluhin’s [27] hypothesis indicates that the specific electric conductivity of the composite and its specific electric resistance depends on the specific electric resistance of the filler and the specific electric resistance of the composite material and is equal to the specific electric resistance of its conditional elementary cell. According to Lopanov’s research [28], a change in electric conductivity activation energy is caused by an increase in the number of contacts between particles.

No comprehensive overview was found in the available sources of the comparison of the effect of shape and granularity of the filler on the conductivity characteristics that would be measured in identical conditions. The goal of this paper is to compare the behavior of the composites in relation to the structure and the genesis of the graphite used, depending on whether the graphite in question is natural or artificially produced, as well as the comparison of the resulting properties affected by the use of fillers with various granularity of the particles (fine, coarse), or possibly, whether it is a entirely natural graphite or with modified properties. Furthermore, a comprehensive comparison of the properties of these materials in relation to the structure of the material, e.g., whether it is a paste or a composite, was not executed. 

The main aim of this research is the modification of the entry components of the cement composite, the matrix of which essentially forms the dielectric, while achieving maximum thinness of the transitional layer of the individual particles. The minimization of the resistance on the interface of the matrix from the dielectric to the conductive filler then could decrease the overall resistance of the composite. In a real application, the conditions in the environment are not constant. Other authors note the significant effect of humidity and temperature of the specimens on the conductivity properties of conductors as well as semiconductors [40,41]. Therefore, it is necessary to monitor the conductivity of the specimens under various conditions. To compare the conductivity properties of materials, the humidity of the environment in which they are placed is not critical but the humidity of the actual specimens. During drying, the specimens dry unevenly in the cross-section. The edge layers dry faster than the core. The currently available sources offer no dependency between the electrical properties of the cement composites and their humidity. Plugin [26] studied the effect of 100% water saturation of the composite and came to the conclusion that it provides a twentyfold reduction of its electrical resistivity to 2.8 Ω·m. A partial goal of this study is the establishing of the effect of humidity on the material and its conductive electrical properties at different levels of water saturation, while using various types of graphite. 

## 2. Materials

The newly designed material with lowered electrical resistivity is based on a silicate. The primary binding component is created by cement (CEM I 42,5 R). The filler component is formed by silica sands and limestone. The conductive component is formed by various types of graphite that differ in their genesis (natural, artificial), as well as their granulometry (coarse, fine). A plasticizer was used to improve the processing of the mixture and decrease the amount of water added. The properties of the entry materials are covered in detail in the following chapters. In order to compare the properties of cement paste and cement composite, specific mixtures were designed. These mixtures were designed with respect to ensuring satisfactory mechanical properties of the silicate even after the addition of high graphite content.

### 2.1. The Bonding Agent

Cement paste and silicate composites were used as binding agents. Portland cement CEM I 42,5 R (according to EN 197-1 [89]) from the cement plant Českomoravský cement, a.s., Mokrá, Czech Republic was chosen, as it is composed of Portland clinker (95–100%) and gypsum (5–6%) that serves as a regulator of the curing process here [89].

The basic properties of the CEM I 42,5 R cement are summarized in Table 1, including selected properties significant for this study [89].

### 2.2. Fillers

#### 2.2.1. Silica Sands

Aggregates according to EN 12620 [90]—aggregates suitable for concrete (ratio or quartz over 95%). The aggregates were supplied by the producer Provodínské písky a.s., Provodín, Czech Republic. Grain size was selected from 0.1 to 4.0 mm. Sands PR 1.6-4, PR 30/31 and PR 35 (commercial names of the products) were selected. The properties and specifications of the selected sands are shown in Table 2 [90].

#### 2.2.2. Limestone

Finely pulverized limestone VBS 40 (commercial name of the product), from the company LB Cemix s.r.o. from the production facility Kotouč Štramberk, Štramberk, Czech Republic, was selected for reference mixtures. Thanks to its granulometry, the VBS 40 limestone is suitable for concrete. Selected properties of this limestone are shown in Table 2.

### 2.3. Admixture

STACHEMENT 2180.1 (commercial name of the product) a super-plasticizing water-reducing admixture on the basis of poly-carboxylate from the producer and supplier STACHEMA CZ s.r.o., Kolín, Czech Republic, was used in accordance with the norm EN 934-1 [91], as shown in Table 3 [91].

### 2.4. Primary Conductive Fillers

Graphite powders from the supplier Epinikon a.s., Vodňany, Czech Republic, were selected as the primary conductive fillers. Both artificial and natural types were selected in various fractions, while both the finest and the coarsest types were included. These graphite powders have different content of carbon and some, according to the producer’s information, have improved electric conductivity properties. The graphite powders used are classified in Table 4.

#### 2.4.1. Supragraphite C300

**Figure 1:**
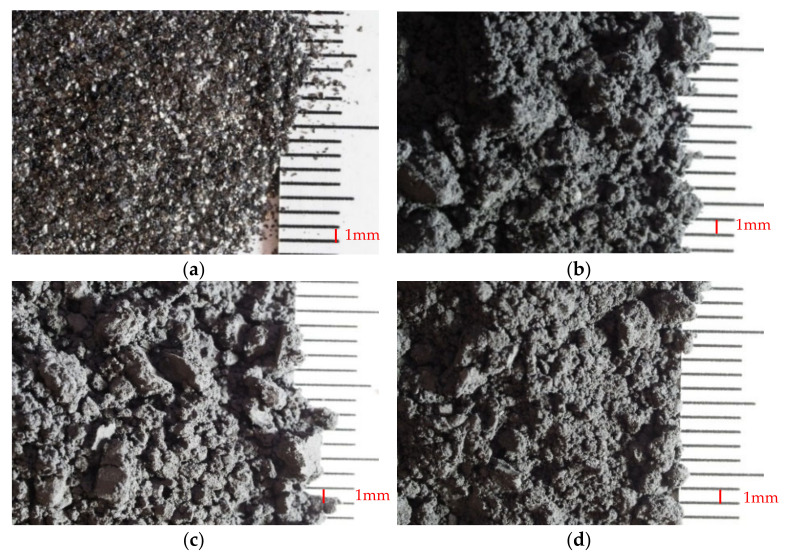
Structure of conductive fillers: (**a**) Supragraphite C300; (**b**) Micrographite UC4; (**c**) Micrographite C4; (**d**) Condufit C4.

Supragraphite C300 (Figure 1a) is a flake-shaped natural graphite. This graphite powder is composed of a minimum of 99.5% carbon and a maximum of 0.5% of ash. The maximum humidity is 0.5%. The granularity, as indicated by the producer, is 60–80% of particles over 100 μm in diameter.

#### 2.4.2. Micrographite C4

Micrographite C4 (Figure 1b) is a ground natural graphite. This micro pulverized graphite powder is composed of at least 99.5% carbon and a maximum of 0.5% of ash. The maximum humidity is 0.5%. Granularity indicated by the producer is 50% of particles within the diameter of 3.5–5.0 μm.

#### 2.4.3. Condufit C4

Condufit C4 (Figure 1c) is a micro pulverized natural graphite with enhanced electrical conductivity properties. This micro-ground graphite powder is composed of a minimum 99.5% of carbon, maximum 0.5% of ash. Maximum humidity is 0.5%. The granularity indicated by the producer is 50% of particles of a diameter ranging between 3.5 and 5.0 μm. The improved electrical conductivity property is achieved by treating the surface of the grain with nanoparticles.

#### 2.4.4. Micrographite UC4

Micrographite UC4 (Figure 1d) is a synthetic micro-pulverized graphite powder. This micro-ground powder is composed of at least 99.0% carbon and maximum of 1.0% of ash. Its maximum humidity is 0.5%. Particle size indicated by the producer is between 3.5 and 5.0 μm.

Properties of the above-listed graphite powders are shown in Table 5 and Table 6.

**Figure 2:**
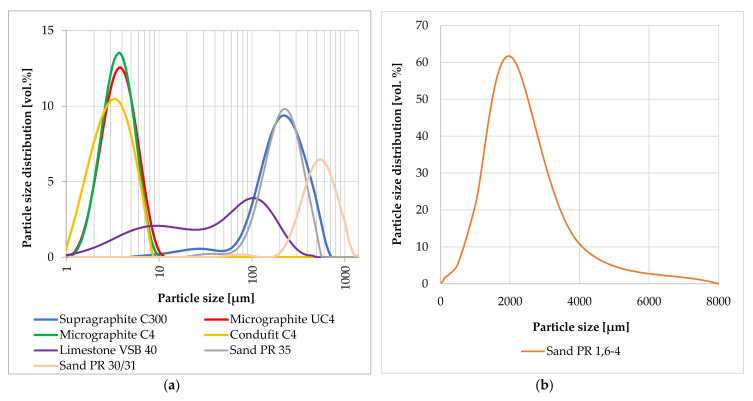
Distribution curve of materials used with particle size: (**a**) below 1 mm; (**b**) above 1 mm.

Figure 2a,b shows the comparison of the particle sizes in a graphic depiction. The distribution curve was established separately for particles under 1 mm and over 1 mm. A Malvern Mastersizer 2000 device (with wet dispersion unit; as a dispersant was used propan-2-ol; the particle dispersion was by sonication unit performed) was used for finer materials (under 1 mm), as it is more suitable for measuring fine materials such as graphite. A test using sieve analysis was used for materials with particle size over 1 mm. As is apparent from Figure 1a, graphite Micrographite C4, UC4 and Condufit C4 type are significantly finer than other materials. Upon mutual comparison of these fine types of graphite, it was found that Condufit C4 contains the smallest particles. Micrographite C4 and Micrographite UC4 have very similar particle distribution; however, Micrographite UC4 contains slightly larger particles. The distribution curve of the coarse-type of graphite Supragraphite C300 is comparable with the finest sand PR 35. Limestone has a rather broad distribution of the curve and contains particles from very fine to approximately 0.3 mm. The measured granularity of various graphite types corresponds to the parameters declared by the producer.

**Figure 3:**
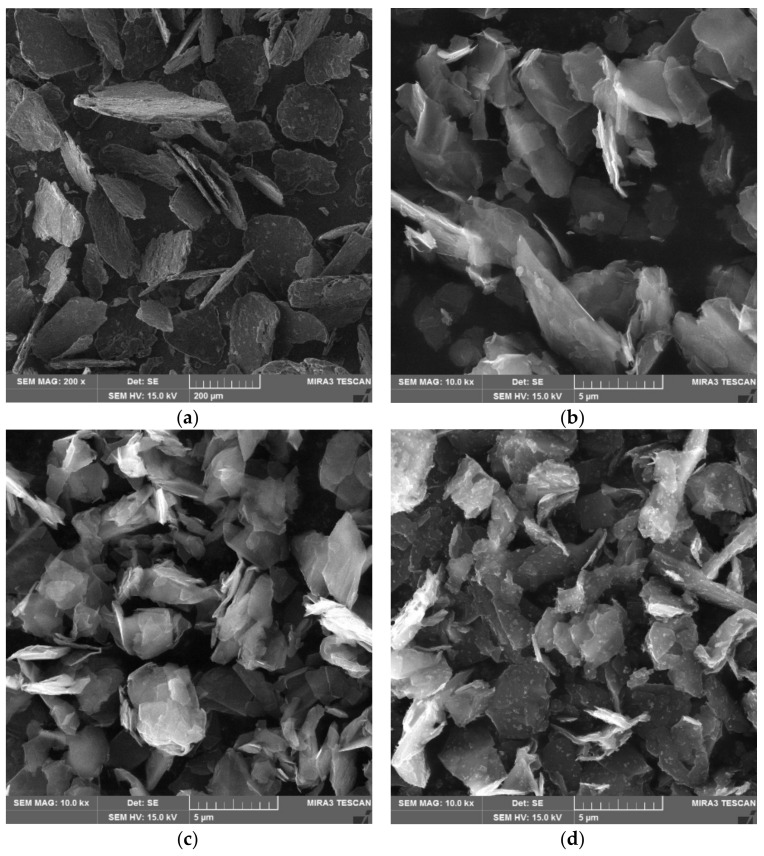
The microstructure of fillers used: (**a**) Supragraphite C300 magnification 200×; (**b**) Micrographite UC4 magnification 10,000×; (**c**) Micrographite C4 magnification 10,000×; (**d**) Condufit C4 magnification 10,000×.

For the particles of graphite, the composition is based on clusters of carbon atoms layered in hexagonal aromatic lamellas, which are apparent from Figure 3. The magnification of the Supragraphite C300 particles (200×) was modified for improved visibility of the particles. The fine graphite types were magnified by 10kx. Thus, the similar structure of platelet shapes became very apparent. The Condufit C4 graphite is declared as a graphite with higher conductivity, additionally containing carbon-based (supplier’s information) nano-ratios visible on the surface of the particles. Images of the Micrographite C4 and of the Micrographite UC4 confirm the results of the evaluations of granularity, where the larger particles are apparent on the Micrographite UC4 image.

It is assumed that the water absorption capacity of conductive fillers will not significantly affect the amount of water added to mixtures and thus of the overall processability of the mixture. The water absorption capacity of the fillers may as a consequence affect the porosity of the composite that increases with the increased amount of used water in the mixture. The sands used to have the lowest water absorption capacity and from the conductive fillers, the coarse Supragraphite C300.

The resistivity of the entry materials was established. In comparison with non-conductive materials such as cement or limestone, the electrically conductive fillers have a lower specific resistance by as much as six orders of magnitude. The resistivity of the conductive fillers ranges in the order of units of Ω·m, while cement and other non-conductive materials are in units of tens of thousands up to millions of Ω·m.

The specific surface area of the particles of conductive fillers relates to their high water absorption capacity and will continue to be monitored regarding whether it will also affect the values of resistivity of the composite. 

The size and type of particles will particularly affect the densification of the mixture and its ability to conduct an electric current. It is necessary to create an even and electrically connected structure from the particles of conductive fillers within the mixture, which could be affected by the type of particles. For this reason, the material selection included irregular, as well as flat or fiber-like particles.

### 2.5. Formulae

#### 2.5.1. Cement Paste

To maximally simplify the verification of the electrically conductive properties of primary conductive fillers in the silicate composite, these fillers were implemented into a separated cement paste. Always 20% of the weight (30% of volume) of the cement in the paste was replaced by a conductive filler, and limestone was used as an example of a non-conductive filler for comparison. The amount of 20% of the weight was selected with the expectation of safely exceeding the percolation threshold, which is usually around 10–12% of weight for graphite fillers [9], while for coarse types of graphite, the percolation threshold may exceed 15%. The resistivity of the composites with the content of fillers beyond the percolation threshold is subsequently more comparable, as it no longer depends on the amount of the filler but on its character properties. The mixture was always mixed while maintaining identical processability. The processability was fine-tuned using spilling on the vibration table according to standard EN 1015-3 [92], with the required spill of 150 ± 10 mm. The composition of the cement mixtures is shown in Table 7. 

#### 2.5.2. The Cement Composites

**Figure 4:**
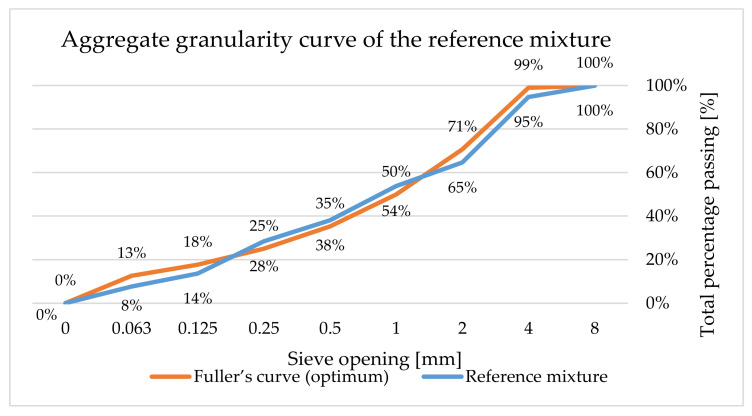
Granularity curve in the reference mixture.

Another step was the verification of the electrically conductive properties of conductive fillers in the cement composite. The purpose of this experiment was the verification of the effects of the used non-conductive aggregate particles on the conducting electric current. The reference mixture was designed as a silicate composite with fine aggregates, fine filler and plasticizer. Portland cement CEM I 42,5 R was selected as the bonding agent. Additionally, three types of sand were used, with the overall distribution of particles between 0 and 4 mm. VBS40, a finely pulverized limestone, was used as the filler, along the plasticizer STACHEMENT 2180.1. The optimum granularity curve was achieved using suitable combinations of the individual aggregates and the filler, to maximally adhere to the ideal granularity curve according to Fuller, 20% of weight of the reference mixture was replaced with the conductive filler as an admixture (see Figure 4). The mixture was also moistened to the same processability that was verified according to standard EN 1015-3 [92]. The required spill was 150 ± 10 mm. Table 8 shows the composition of the reference mixture and the mixtures including the conductive composite.

The composition of the reference composite was designed to achieve high compressive strength, approximately 50 MPa after seven days. The reference mixture was intentionally designed to ensure satisfactory strength even after the addition of the conductive fillers.

## 3. Methods

The following set of methods was selected for the study of the impact of the individual conductive fillers and their interaction with other materials in the cement paste and the composite. These are methods focusing on the description of materials from the perspective of physical and mechanical parameters, as well as the perspective of one of the most important properties—resistivity. In the case of produced cement pastes and composites, the basic parameters are again resistivity and strength and, in particular, the analysis of the structure using an optical microscope and a scanning electron microscope.

### 3.1. Loose Bulk Density of the Fillers

The bulk density of the loosely poured aggregate was established using the standard EN 1097-3 [93]. A vessel of 1 L volume was used, suitable for aggregate up to D_max_ = 4 mm. The weight of the aggregate compacted by vibration was stipulated in accordance with the standard EN 1097-3 [93], similarly to the loose bulk density of poured aggregate with the difference that the sample was subsequently compacted by vibration on the vibration table. The measurement was performed on a scale with a precision of a hundredth of a gram.

### 3.2. Volumetric Density

Volumetric density as established using pycnometric methods, the measurement was carried out in technical alcohol due to insufficient wettability of the conductive fillers. The entire measurement was in accordance with standard EN 1097-6 [94]. 

### 3.3. Specific Weight

Specific weight was identified using the helium AccuPyc II 1340 Pycnometer. This pycnometric method used inert gasses, such as helium or nitrogen, to establish specific weight. This method is more precise than the existing method using water or alcohol as the measuring medium. The specific weight is calculated from the ratio of weight and volume.

### 3.4. Establishing the Distribution of Particles and Sieve Analysis

Granulometry was established for the conductive fillers. In the case of the coarse graphite (Supragraphite C300) it was determined using sieve analysis in accordance with standard EN 933-1 [95] on a standardized set of sieves. 

In the case of the other conductive fillers, due to their highly fine structure and character of particles, their granulometry was established using the method of laser diffraction analysis according to standard ISO 13320:2009, on device Malvern Mastersizer 2000. The principle of this method lies in a continual stream of sample particles lit by a laser beam, which subsequently bends (refracts) or falls apart (diffracts). The bent beams subsequently form a diffraction image that is captured using a Fourier lens. Using Fourier’s transformation, this image is then turned into a granularity curve on a computer. The advantage of this method is particularly the ability to establish the ratio of very fine particles and the possibility of dividing the curve according to fractions of our choice. 

### 3.5. Specific Surface

The specific surface of the conductive fillers was determined using the BET method according to standard ISO 9277:1995 (E). This method uses a single-layer or multi-layer physisorption of nitrogen gas molecules onto the surface of the tested material at lowered pressure. The volume of the adsorbed gas is subsequently evaluated. 

### 3.6. Material Water Absorption Capacity

The water absorption capacity was only established in the case of conductive fillers and Supplementary Materials that are not common components of silicate composites. Because the conductive fillers are very fine powder materials, their water absorption capacity is established using a measuring set with a Büchner funnel and vacuum pump according to standard EN 13055 (D) [96]. The water absorption capacity is identified as WA(t), where “t” expresses the time for which the sample is left saturated. The measurement used the common time of t = 5 min.

### 3.7. Determination of the Resistivity of Materials

The resistivity of the actual powder materials is one of the most significant properties of conductive fillers, as well as the other entry materials, which can help us select the most suitable materials to achieve optimal electrically conductive properties. The resistivity of materials depends on their chemical composition, surface treatment, their ability to compress and the character of the particles of the tested material. From this perspective, resistivity is only tested in powder materials.

Because prescribed standards and processes for this type of measurement are not available, a brand new methodology was created to verify the resistivity of materials. During the development of the methodology, emphasis was placed on simplicity and repeatability of the measurement. New measurement forms were produced using 3D printing that were subsequently connected with devices for the measurement of impedance. Subsequently, the resistivity of the material was determined.The prepared device with electrodes was partly filled up to approximately 90% of its capacity with the material. The sample was subsequently compressed using the pressure of 100 N using a press (pressure 1.67 N/mm^2^). Using a table measuring device GW Instek LCR-6020, the impedance of the material was established and subsequently resistivity was calculated.

**Figure 5:**
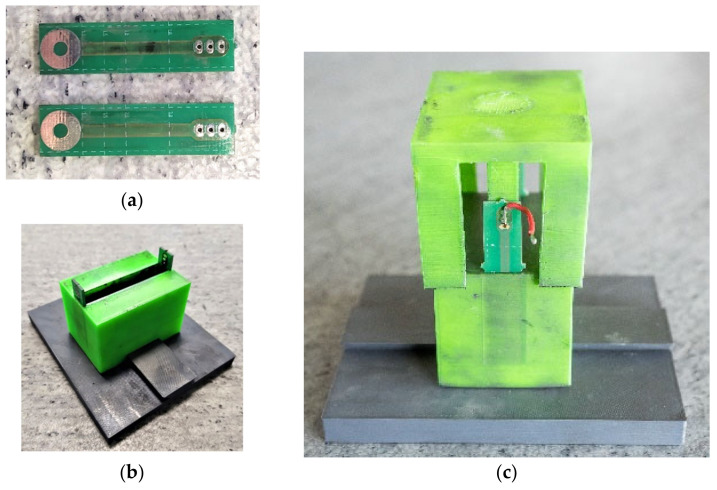
(**a**) A detailed photograph of the electrodes; (**b**) detail of the chamber with inserted electrodes; (**c**) device prepared to achieve the compression pressure, including its upper pared and subsequent measuring of impedance.

The device for measuring impedance (Figure 5) was created by a 3D printer using a non-conductive plastic PETG. The measuring chamber is 10 mm wide, 60 mm long and 50 mm deep. Two opposing electrodes were inserted into the device, 60 mm apart, which are, upon compression of the material, subsequently attached to a measuring device.

The GW Instek LCR 6020 measuring device measures impedance directly in (Ω) and the phasor angle in (°), describing the phase movement between the voltage and current. This device will be subsequently used to determine the impedance of test specimens, and the impedance is then calculated for a specific resistance, i.e., resistivity.

### 3.8. Determination of the Processability of the Fresh Mixture

The processability of mixtures is determined using a vibration table that is commonly used to determine the consistency of fresh mortar. A standardized truncated cone measuring 60 mm, with a bottom internal diameter of 100 mm and internal upper diameter of 70 mm, including the funnel, was used in accordance with standard EN 1015-3 [1].

The spill of 150 ± 10 mm was determined as a suitable and firm processability. 

### 3.9. The Production and Storage of Test Specimens

First, all dry components were doses and homogenized together in a mixer for the duration of 2 min. Added water was gradually dosed to moisten the mixture. Subsequently, the plasticizer was mixed in 50 mL of water to achieve optimum homogenization and added. Then the rest of the water was supplemented to achieve the required consistency. After mixing for the duration of at least 2 more minutes, the desired processability was tested on the vibration table.

**Figure 6:**
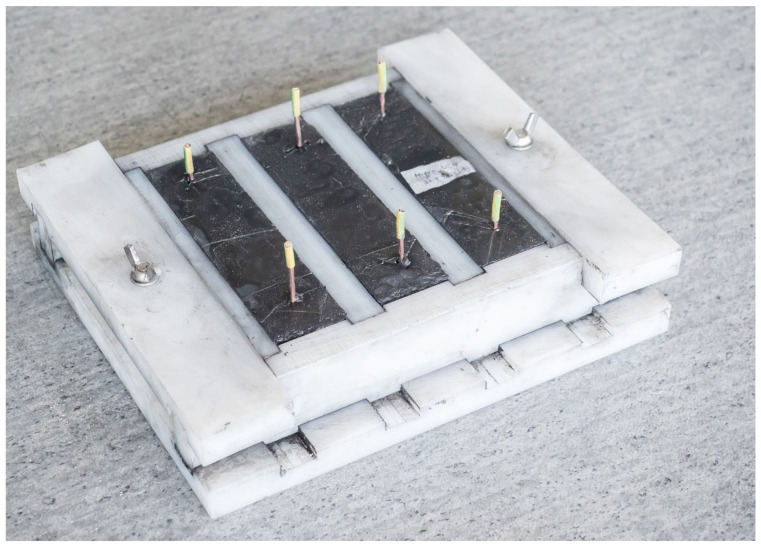
A mold with specimens set with electrodes for the measuring of impedance.

The mixture was then poured into molds of 40 × 40 × 160 (mm) according to standard EN 196-1 [97] (Figure 6) and densified on the vibration table. In the case of the mixture of the composite material, the mixture was densified manually using a flat spatula. After filling, the molds were covered by an air-tight foil to prevent the possible evaporation of added water. Upon the removal of the mold, the specimens were placed in a water environment where they were cured for 7 to 28 days.

**Figure 7:**
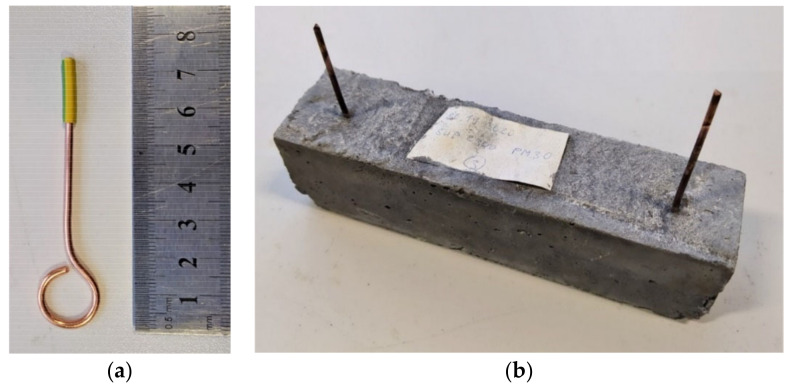
The measuring of impedance: (**a**) a detail of the copper electrode for measuring impedance in test specimens; (**b**) a test specimen with electrodes.

To determine the impedance, testing specimens of the dimensions 40 × 40 × 160 (mm) were produced from the prepared pastes and composite–material mixtures, into which copper electrodes were inserted 12 cm apart and 2 cm from the edges of the closest three sides (Figure 7). Specimens of the dimensions 40 × 40 × 160 (mm) without the electrodes were created for the purpose of testing tensile strength and compressive strength.

### 3.10. Determining the Resistivity of Cured Specimens

The determination of the resistivity of test specimens was carried out using an original methodology. First, impedance was identified for the specimens and subsequently calculated into resistivity. Copper electrodes were incorporated in the specimens during production (Figure 7a), located 120 mm apart (see Figure 7b). There electrodes were produced from a copper wire with a diameter of 2.5 mm, which was shaped into a circle using a device. A total of 15 mm of insulation was left on the electrodes to maintain a clean part for future measurements. These electrodes were inserted into the fresh mixture in a mold using a device created by a 3D printer. This achieved the specific distance of the electrodes of 120 mm and the surrounding area was carefully densified to achieve direct contact with the material.

The impedance was measured after 28 days on samples saturated with water. Subsequently, it was measured in partly dried states and in the end in a completely dry condition. The measurement in the saturated state was carried out upon removal of the specimen from water and subsequent drying-off of the surface. The measurement of the partly dry states of the specimens was carried out gradually over the course of 90 days since production. In case the specimen failed to achieve 50% water content during the 90 days of drying (based on the difference of weight between the dried and fully saturated sample), the drying was continued until this threshold. To measure the impedance in the dry state, the specimens were dried at 90 °C until achieving constant weight.

The advantage of this method of measuring impedance is the assured and direct contact of the material with electrodes, as well as very good securing of the electrodes against pulling out or loss of contact with the material due to volume changes of the material. Device GW Insteak LCR 6020 was chosen for the measuring of impedance.

### 3.11. Verification of Mechanical Properties

Compressive strength and flexural strength were always measured on 3 test specimens in a solidified state after 28 days of curing. The flexural strength was established in the test specimens with dimensions of 40 × 40 × 160 mm using a three-point bending with a 100 mm span between supports according to standard EN 12390-5 [98]. The compressive strength was measured using 6 fragments of the specimens left after establishing flexural strength, in accordance with standard EN 12390-4 [99].

### 3.12. SEM (Scanning Electron Microscope)

The surface structures of the conductive filler particles and the structures of the created cement pastes and composites were analyzed using a scanning electron microscope. This technology uses a narrow cluster of electron beams produced in an electron jet under the tension of 0.1–30 kV. The specimen as well as the beam are in a deep vacuum. A wolfram jet was used with a voltage of 15 kV. Upon the stream of electrons hitting the specimen, various interactions occur that are captured using detectors and subsequently evaluated by a computer. The output is a black-and-white image of the surface of the particles. This technology enables magnification up to 1,000,000×. For our purposes, a 200× magnification was used for the coarse type of graphite Supragraphite C300 and a 20,000× magnification of other types of graphite, since the size of the particles is fundamentally different. The analysis of the structure of the cement pastes and composites was carried out on fracture surfaces. Polishing the cut caused carbon particles to spread across the polished surface and prevented the execution of the EDX analysis with relevant results.

### 3.13. EDX (Energy Dispersive Analysis)

The energy dispersive analysis of the characteristic X-ray radiation is a non-destructive method intended for the determination of the local composition of the examined material. The characteristic roentgen rays are formed by the interaction of an electron ray focused into a circle of a diameter of up to several tens of nanometers. The individual components are then characterized on the basis of energy necessary for the transition between the energy levels of electrons. This method was newly employed in the study of the microstructure of electrically conductive composites on the basis of graphite, using a local identification of the material composition of the specimen. This method enables color-differentiation of conductive particles from the surrounding non-conductive matrix. It is possible to visually present the distribution and also the internal structure of the specimens, the distribution and mutual contact of the particles that subsequently form the electrically conductive network. Thus, this method appears very suitable for research in the area of microstructures of electrically conductive composites on the basis of carbon.

### 3.14. Optical Microscopy

The specimens were tested from the perspective of the degree of even dispersion of the filler component in the polished cross-sections (pastes) and unpolished cross-sections (composites). Polishing of composite samples was not possible due to damaging of the surface by the releasing sand particles. A digital optical microscope with a 2500× lens was used for the determination of the microstructure, as it is capable of achieving a magnification equivalent to approximately 800× of an optical microscope. The device uses an optical system and a digital camera with a high resolution, equipped with an optical cable for an immediate transfer of the captured image to a computer. Due to the different depths of focus of the individual scanned layers, the technology of a composite image was used to achieve maximum possible sharpness. A polarizing aperture was used for the differentiation of the carbon particles. This uses the fact that light is in its essence an electromagnetic wave that oscillates on all levels that are perpendicular to the direction of the ray. The insertion of the polarizing screen into the path of the light refracted from the surface of the sample creates a polarized ray. Upon interference, the recombined ray has different properties depending on the optical properties of the double-refraction object and the slight turning of the analyzer. The record of the recombined ray with an appropriately turned analyzer enables the clear distinction of spatially oriented particles of carbon in the specimen, as they have a different refraction index from the matrix.

## 4. Results

### 4.1. The Verification of the Electrically Conductive Properties of the Cement Paste, with a Graphite Filler

**Figure 8:**
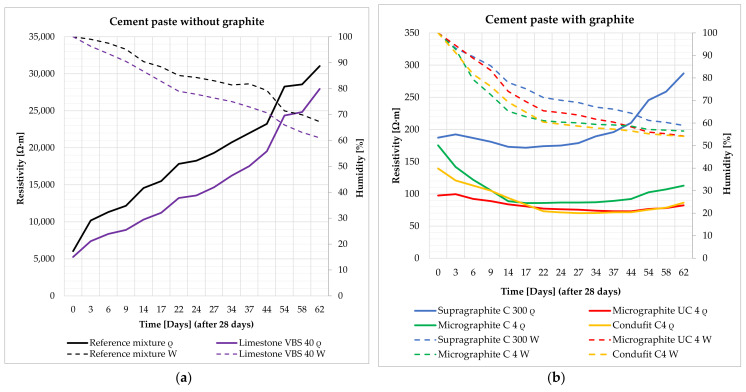
The effect of humidity on the resistivity of cement pastes: (**a**) without the graphite; (**b**) with the graphite.

**Figure 9:**
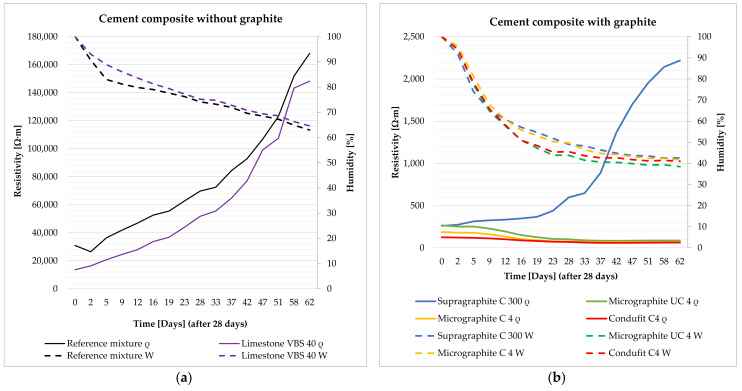
The effect of humidity on the resistivity of silicate composites: (**a**) without the content of graphite; (**b**) with the content of graphite.

**Figure 10:**
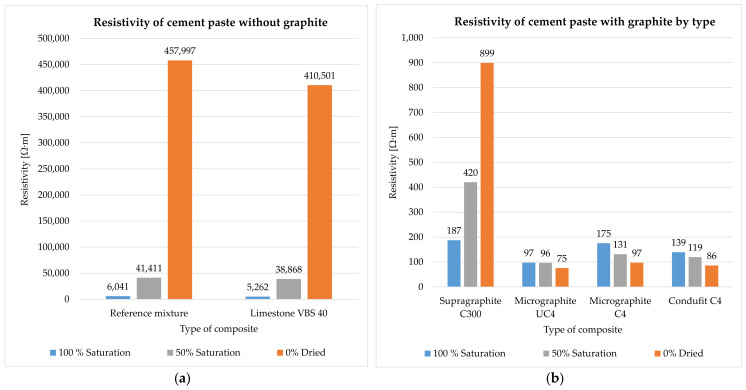
Comparison of resistivity in cement pastes at 100%, 50% and 0% water saturation, samples: (**a**) without the content of graphite; (**b**) with the content of graphite.

**Figure 11:**
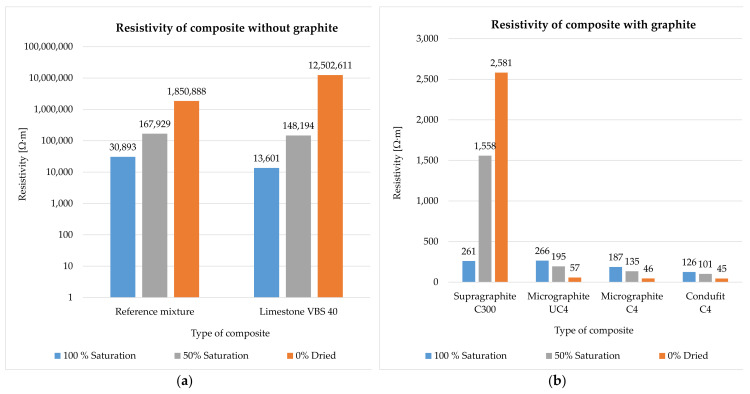
The comparison of resistivity of silicate composites at 100%, 50% and 0% water saturation, samples: (**a**) without the content of graphite; (**b**) with the content of graphite.

The impedance was measured and subsequently calculated into resistivity after 28 days of curing of the specimen in water storage (water temperature 20 ± 2 °C). Their humidity thus corresponded to 100% water saturation. Subsequently, the specimens were left in a laboratory environment (20 ± 2 °C, 50 ± 10%) and dried in open space. The impedance and humidity of the specimens were measured during the gradual drying for the duration of 62 days (90 days from creating the mixture) (Figure 8, Figure 9, Figure 10 and Figure 11). The determination of the impedance was executed on specimens with dimensions 40 × 40 × 160 (mm) (Figure 7).

The cement paste without conductive fillers acts as a dielectric, meaning an insulating agent, capable of polarization (Figure 8a). The resistivity of the cement paste primarily depends on the water content, while the decrease in contained water by 10% increases resistivity by approximately 2×. Further, the density also has an effect on the resistivity of the cement paste. The denser and more integrated the structure of the paste is, the closer the atoms are and the higher likelihood the electrons have of conducting a flashover. This effect was achieved by the addition of fine limestone VBS40 to the paste, as a fine admixture that fills the structure. The contained water in the cement paste, without conductive fillers, supplies the paste with free ions that subsequently decrease resistivity.

In the case of the cement paste, after the addition of electrically conductive fillers on the basis of graphite, there is a significant decrease in resistivity in both the 100% saturated state and in the gradual decrease in water content (Figure 8b). This is where the difference from the cement paste without conductive fillers is the most significant. Resistivity decreased up to 50× in the saturated state with the introduction of graphite into the cement. Upon gradual decrease in water content in the cement paste, there is a mild decrease in resistivity, as water has higher resistivity than the graphite mixture. The coarse type of graphite, Supragraphite C300, shows the highest values of resistivity, resulting from the lower ability of the coarse particles to create an integrated structure. To a certain degree, Supragraphite C300 uses freely bound water as an electrolyte for the conducting of electrical current through a cement. With the reduction of water under 80%, the resistivity of the test specimens begins to rise. In the case of the finest type of graphite, Condufit C4, the lowest values of resistivity were obtained upon gradual decrease in the saturation of the specimen with water. Overall the lowest values of resistivity were reached. The low values are caused by the very fine granularity and nanoparticles that increase the contact of the individual particles, thus helping to conduct the electric current. Graphite Micrographite UC4 and C4 have nearly identical parameters and only differ in their genesis and partly in granularity. As is apparent from the above graph, natural Micrographite C4 is more effective and consistently achieves lower and constant resistivity values than the synthetic graphite with the content of larger particles.

The composite without a conductive filler acts the same way as the cement paste alone (Figure 9a). The contained water provides the composite with free ions for the transmission of electric current. With the decreasing water content in the composites, the resistivity increases significantly, just as in the case of the cement paste alone. Unlike in the case of the cement paste as such, the composite has up to 5× higher resistivity thanks to the incorporated non-conductive fillers, such as the mixture of silica sands and limestone. As in the case of the cement paste, with the addition of fine limestone, and also in the case of composites, the structure is filled in and shows a subsequent decrease in resistivity as opposed to the reference mixture.

The composite with the content of graphite shows the same trend of the cement paste with graphite. With the addition of graphite to the composites, the resistivity changes significantly (Figure 9b). With the decreasing amount of contained water, the resistivity of composites with a fine type of graphite decreases. In the case of the coarse type of graphite, Supragraphite C300, with the reduction of the contained water, the resistivity increases, caused by the significantly higher granularity of the particles that results in a less perfect interconnection of the electrically conductive network and is further affected by the presence of non-conductive sands. As opposed to the cement paste without the graphite, the resistivity of composites with graphite is higher thanks to the content of non-conductive fillers. Further, this composite is less sensitive to the amount of contained water than in the case of the cement paste. The most stable and lowest levels of resistivity were reached in the case of used filler Condufit C4 with improved electrically conductive properties. In the case of C4 and UC4 graphite, the difference was only notable in the saturated state. Once the saturation with freely bonded water decreased below 50%, almost identical values of resistivity were measured in all composites with fine types of graphite.

According to the trends shown in Figure 10a, it is apparent that in the case of a mixture without conductive fillers, resistivity steeply increases with the decreasing water content. The graph also shows the apparent effect of used limestone as a non-conductive admixture that was intended to fill the structure.

As is apparent from the result (see Figure 10b), it was found that finer types of conductive fillers show lower values or resistivity than coarse fillers, despite coarse fillers significantly decreasing the resistivity in comparison with the reference mixture. The results show that in the case of less conductive and non-conductive fillers, the humidity of the specimens is a very significant parameter. In the case of the cement paste with the content of the coarse type of graphite or in the cement paste alone, upon decreasing the water saturation to 50%, the increase in resistivity is approximately 70× and subsequently, after a complete drying, the increase in resistivity was 750×. The synthetic Condufit C4 has shown very stable properties at various degrees of saturation. Again, the Micrographite C4 shows lower values than the synthetic Micrographite UC4. In comparison with the coarse-type Supragraphite C300, the fine types of graphite reach very similar values.

The effect of humidity in the composites without graphite filler corresponds to the trends of cement pastes. In the reference mixture, upon decreasing the saturation to 50%, resistivity increased approximately 55× and at complete drying it increased by 600×. The differences were even more significant for the mixture with limestone as a filler. Upon decreasing the saturation to 50%, resistivity rose 100× and upon complete drying, by 9200× (see Figure 11a).

The resistivity of the composites with the graphite-based filler performs differently depending on the use of coarse or fine graphite. In the case of the use of fine types of graphite, with decreasing saturation of the specimens with water, their resistivity also decreases. Upon reducing the water saturation by 50%, the resistivity decreased by approximately 30%, and upon complete drying, the resistivity decreased by up to 75%. However, in the case of using the coarse type of graphite, upon the decrease in specimen saturation by 50%, there is a significant increase in resistivity by 60× and upon complete drying by 99×. In the case of the composite with the coarse filler, water is necessary to achieve a low level of resistivity, as it creates suitable electrically conductive bridges between the conductive particles of graphite. In the case of a fine filler, water has higher resistivity values than the actual network formed by fine particles of graphite, thus forming a dielectric insulator. The lowest achieved resistivity values were through the addition of the Condufit C4 graphite with enhanced electrically conductive properties. In the case of the composite with a higher water content, the values of resistivity using Micrographite C4 and Micrographite UC4 reach larger differences than in the case of the paste. However, upon complete drying, the values are comparable (see Figure 11b).

### 4.2. The Physical and Mechanical Properties of Cement Pastes and Composites

The physical and mechanical properties were established according to standards EN 12390-5 [98] and EN 12390-3 [100], using specimens of the size 40 × 40 × 160 (mm) after 28 days of curing in a water environment (water temperature 20 ± 2 °C).

**Figure 12:**
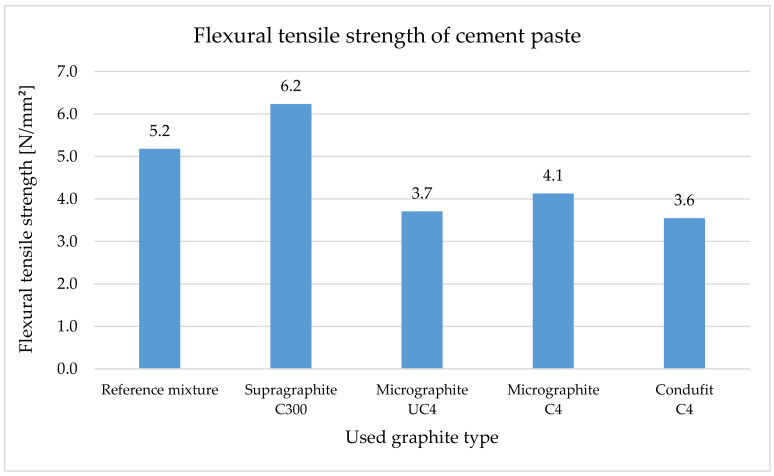
Flexural tensile strength of cement paste.

The flexural tensile strength (Figure 12) increased by 20% when using the coarse type of graphite. This is caused particularly by the shape and size of the particles that transfer load better than the cement paste alone. Upon using the fine types of fillers, in all three cases, the strength similarly decreased. This was approximately a 30% decrease. This was caused by the content of very small particles subsequently forming a large transient zone that is not as homogenous as the cement paste alone. 

**Figure 13:**
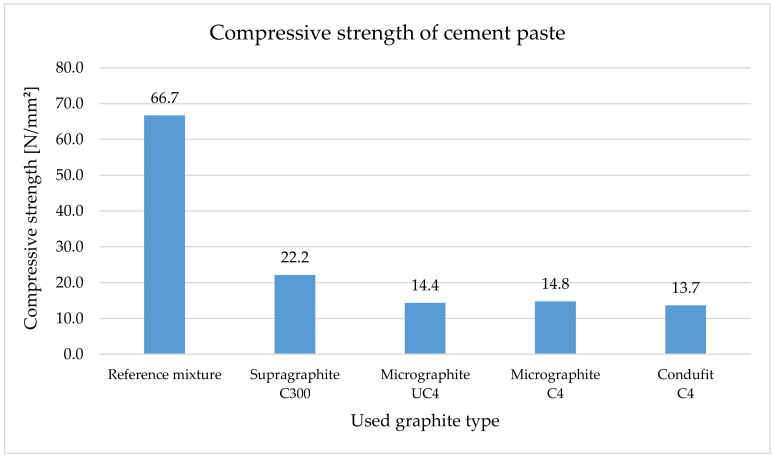
Compressive strength of cement paste.

Compressive strength (Figure 13) decreases significantly with the addition of graphite fillers to the cement paste. The size and shape of the particles also show in the compressive strength. When using the coarse type of graphite, the decrease in compressive strength was 67%, and in the case of fine types of graphite, the comparable decrease was approximately 78%.

**Figure 14:**
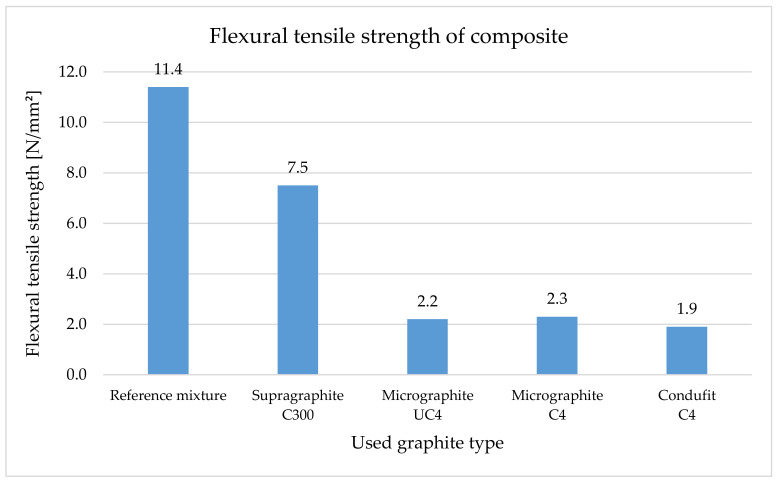
Flexural tensile strength of the composite.

In the case of the composites, the flexural tensile strength (Figure 14) is considerably higher for the reference mixture due to the used aggregate. Upon using the coarse type of graphite, the flexural tensile strength of the composites decreased by 34%. Upon using the three fine types of graphite in the composite, this strength decreased very similarly, approximately by 82%. Unlike in the case of cement pastes, flexural tensile strength in the case of the composite decreases because the composites contain large amounts of fillers that decrease flexural tensile strength. At the same time, less bonding agent is contained here.

**Figure 15:**
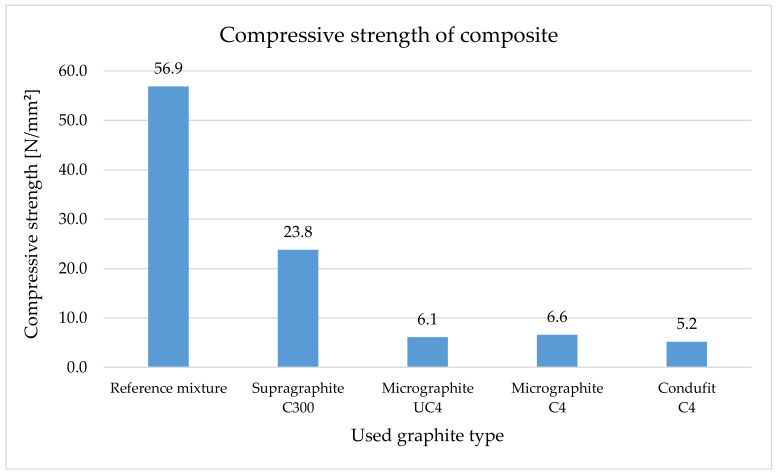
Compressive strength of the composite.

The effect of fine graphite on the compressive strength is more notable (Figure 15), where they decreased the compressive strength by approximately 90%. The addition of a coarse type of graphite caused a lower decrease, by 58%.

### 4.3. The Study of the Structure Using Optical Microscope

The microstructure of the cement pastes, as well as composites, was analyzed using an optical microscope. The structure was monitored on the surface of the cross-sections. Subsequently, a polarization screen was used to highlight and color the differentiation of graphite particles (green) from the matrix (white, grey, brown); in the case of the non-conductive fillers, the internal network of electrically conductive elements and the formed structure was monitored.

**Figure 16:**
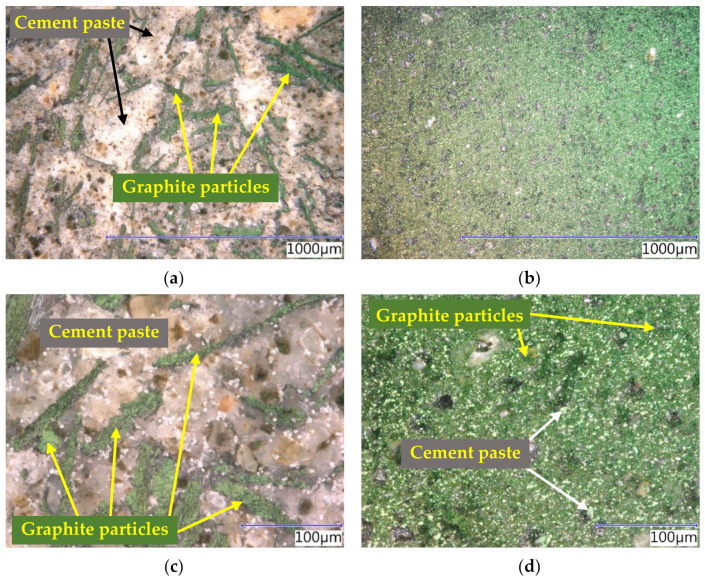

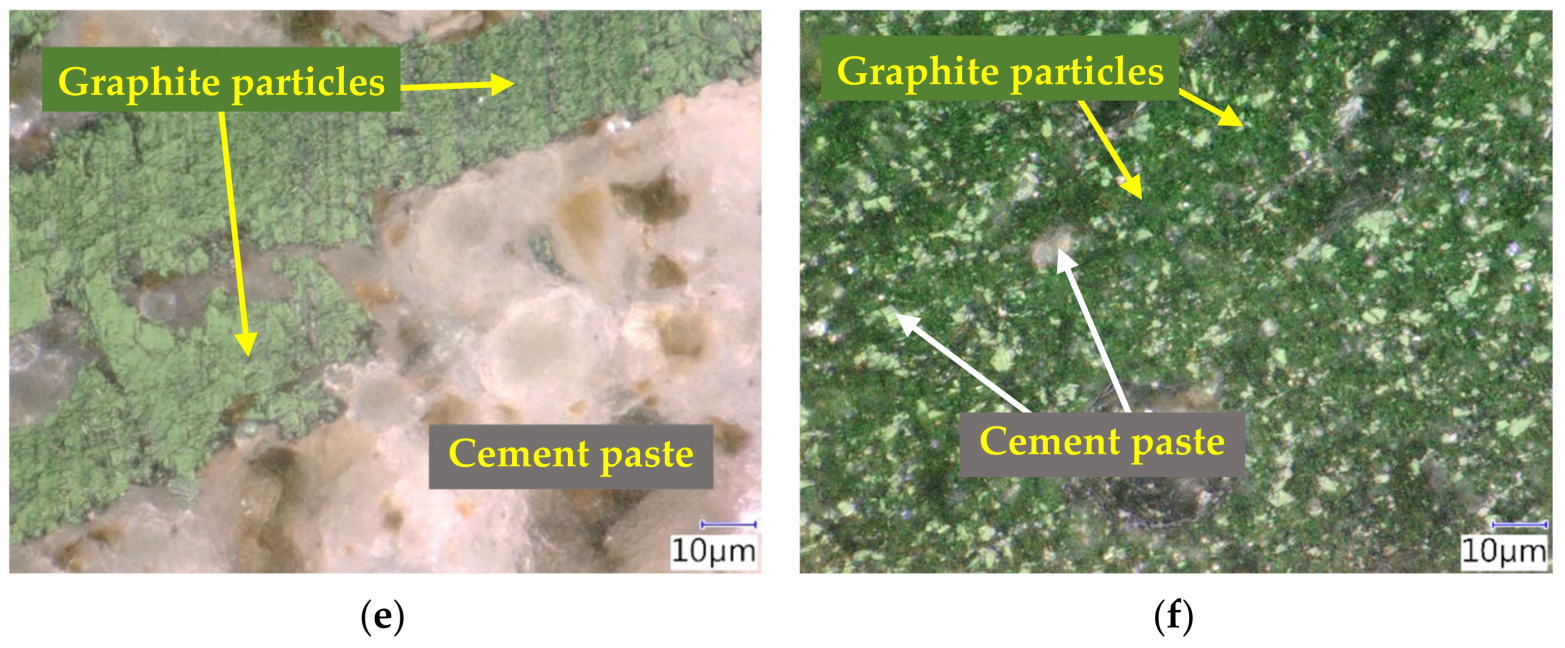
Images from the optical microscope: cement pastes with the graphite filler on the edge (Supragraphite C300—(**a**,**c**,**e**); Condufit C4—(**b**,**d**,**f**)) upon 250× magnification (**a**,**b**), 1000× magnification (**c**,**d**), 2500× magnification (**e**,**f**).

The structure of the cement pastes (Figure 16) was examined to study the internal distribution of the conductive filler within the individual plane of the cut. Upon comparing the structure of the cement pastes with the addition of the coarse Supragraphite C300 and the fine Condufit C4, it is apparent that the structure with the coarse graphite is rather heterogenous, and the individual graphite particles are separated by a thick layer of non-conductive cement paste. This is the main reason for the high resistivity of the mixture. Upon using a fine filler, a homogenous distribution was achieved, with a significant potential to achieve high conductivity. As is apparent from the optical microscope images, the graphite particles are in mutual contact, forming a unified conductive structure that is more suitable for transmitting an electrical charge. This supports the significant differences in the behavior of both types of mixtures in establishing resistivity. 

**Figure 17:**
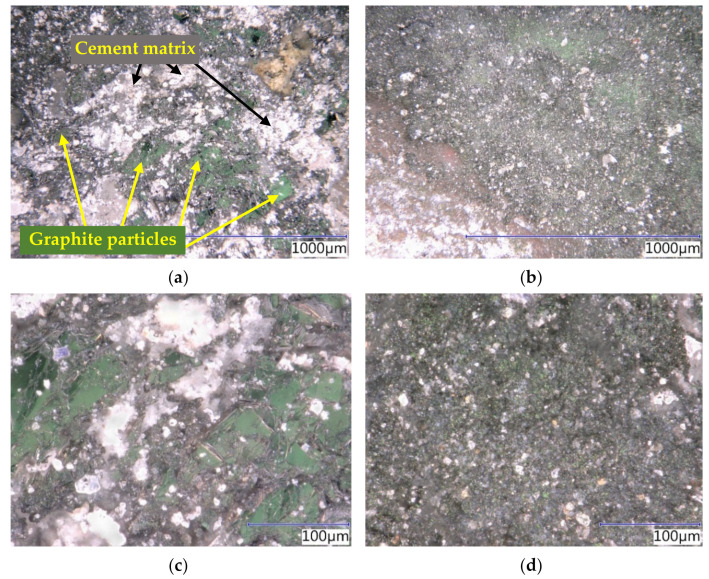

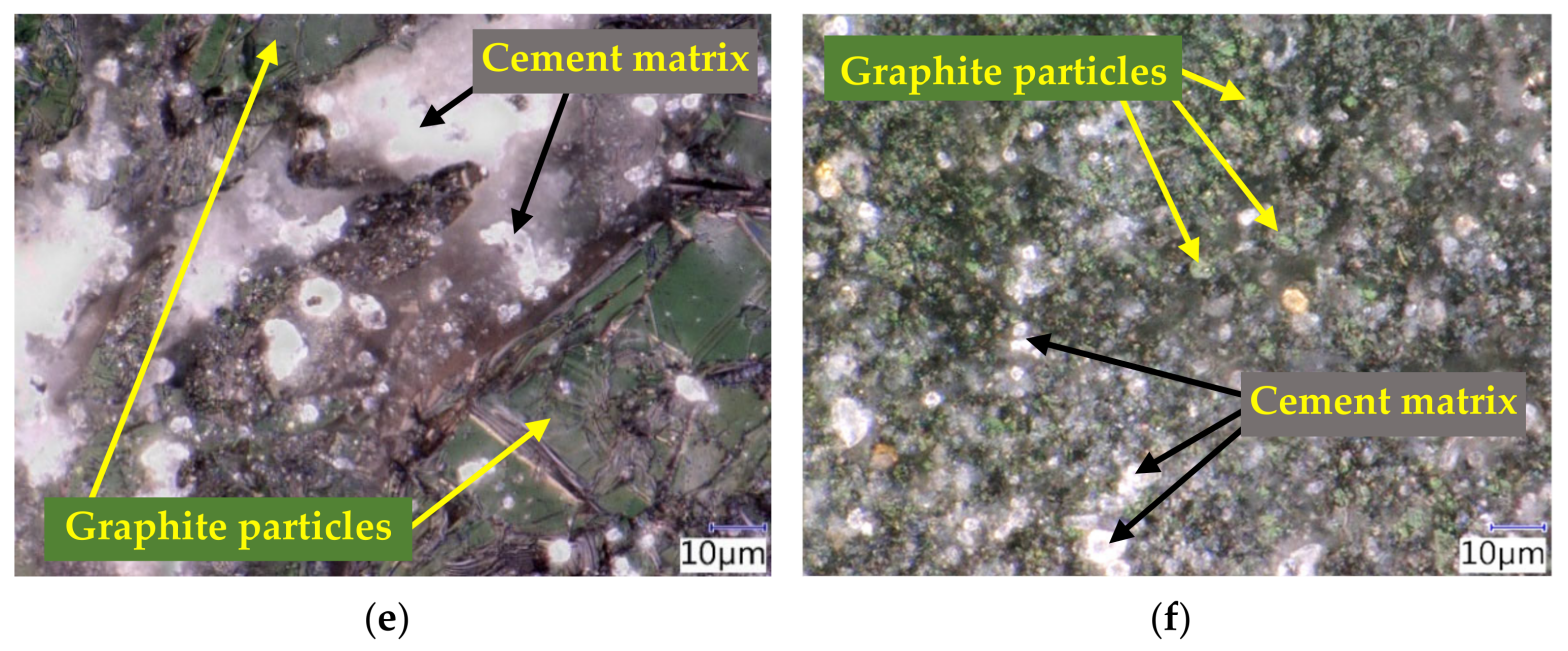
Images from the optical microscope: cement composites with graphite filler in cut specimens (Supragraphite C300—(**a**,**c**,**e**); Condufit C4—(**b**,**d**,**f**)) at 250× magnification (**a**,**b**), 1000× magnification (**c**,**d**), 2500× magnification (**e**,**f**).

The microstructure of the specimens (Figure 17) of the cement composites was studied on cross-sections of the samples. Similar to the pastes, the images show the apparent difference in the contact zone. While the specimens with the coarse graphite (Supragraphite 300) fail to have a perfectly formed connected conductive network, the composites with the fine conductive filler (Condufit C4) have it. This confirms the results established by measuring the impedance. 

### 4.4. The Study of the Structure Using a Scanning Electron Microscope

**Figure 18:**
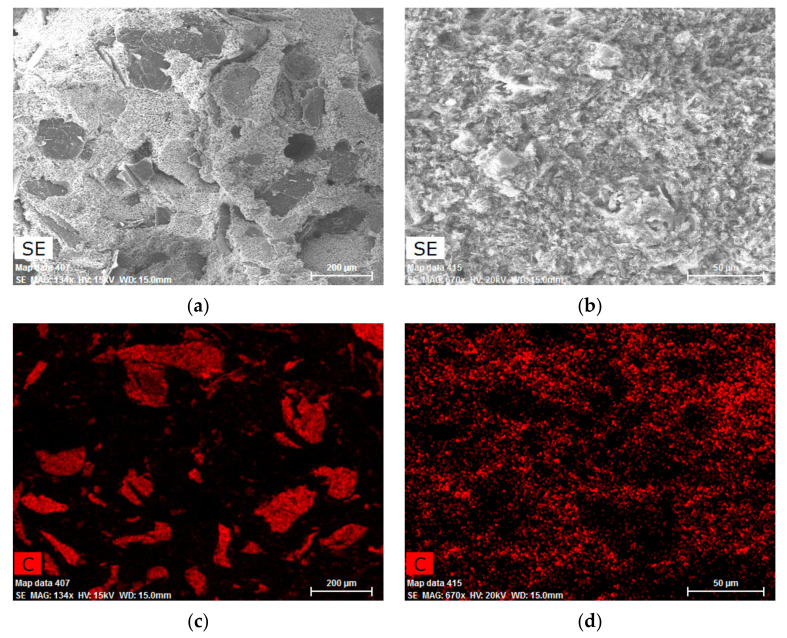

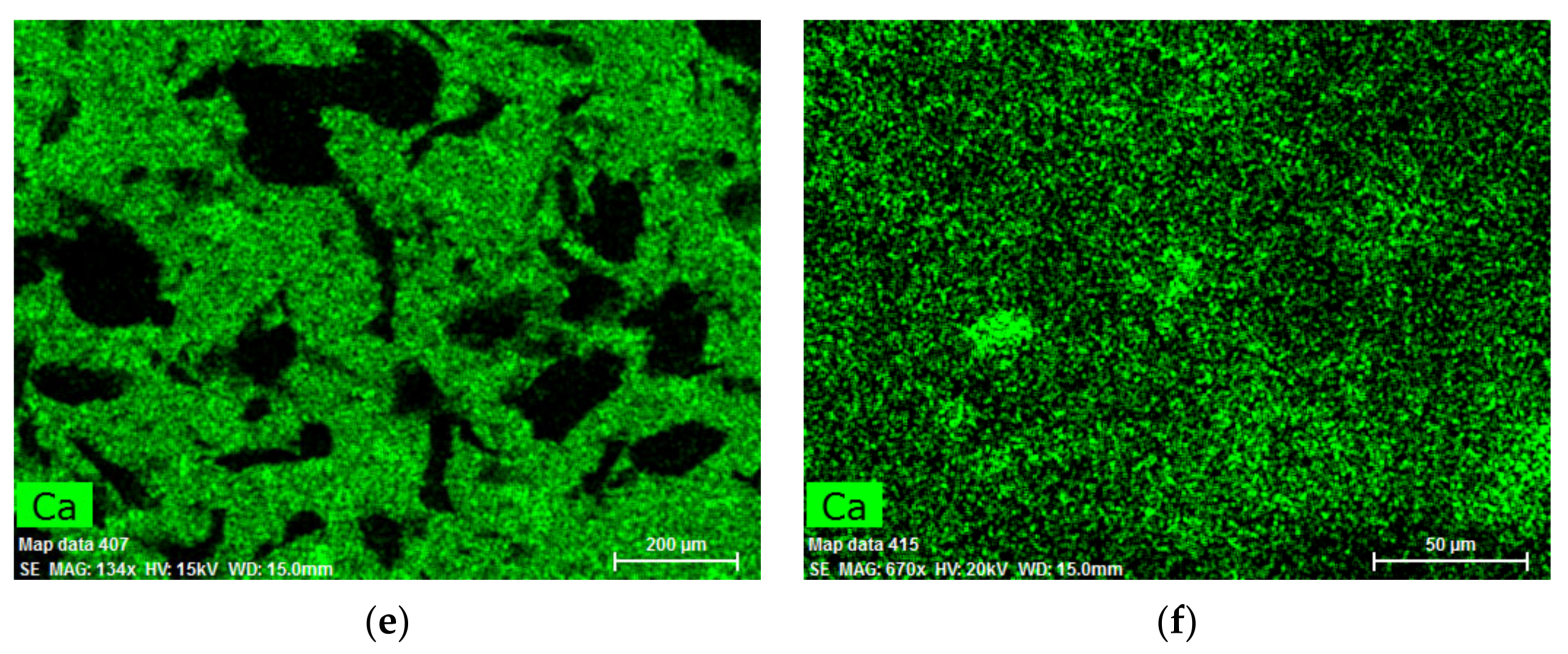
Images from the scanning electron microscope using the EDX analysis of cement pastes with graphite fillers: (**a**) SEM of the surface of a specimen with the Supragraphite C300 filler upon magnification 134×; (**b**) SEM of the surface of a specimen with the Condufit C4 filler upon 670× magnification; (**c**) the red presence of carbon in a specimen with the Supragraphite C300 filler upon 134× magnification; (**d**) the red presence of carbon in the sample with the Condufit C4 filler upon 670× magnification; (**e**) the green presence of carbon in a specimen with the Supragraphite C300 filler at 134× magnification; (**f**) the green presence of carbon in a specimen with the Condufit C4 filler upon 670× magnification.

The evaluation of the images from the scanning electron microscope (Figure 18) while using the EDX analysis clearly shows the differences in the distribution of the graphite filler within the matrix. While the coarse graphite is surrounded by a rather thick layer of non-conductive calcite-containing material, fine graphite creates a homogenous mixture. The areas without the red-marked occurrences of carbon on the images with the fine graphite are frequently depressions that could not be examined using the EDX analysis.

**Figure 19:**
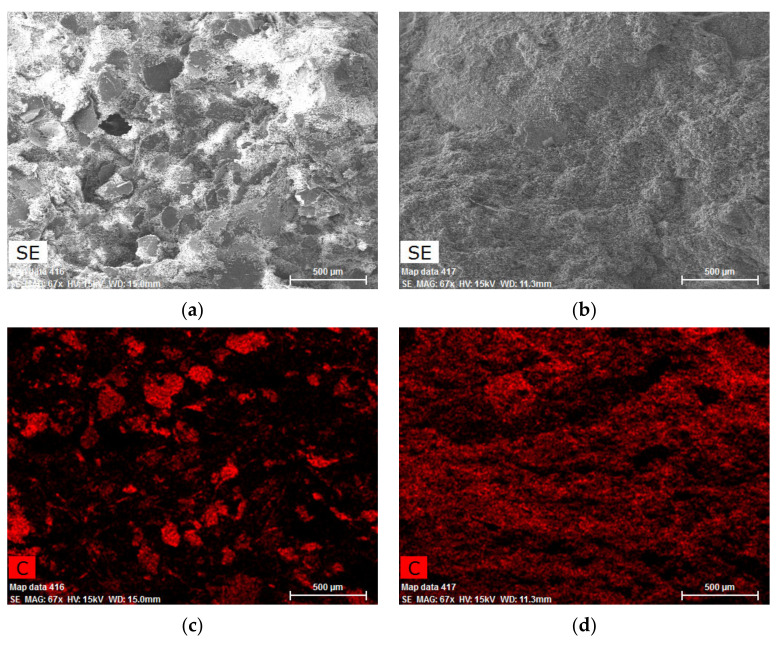

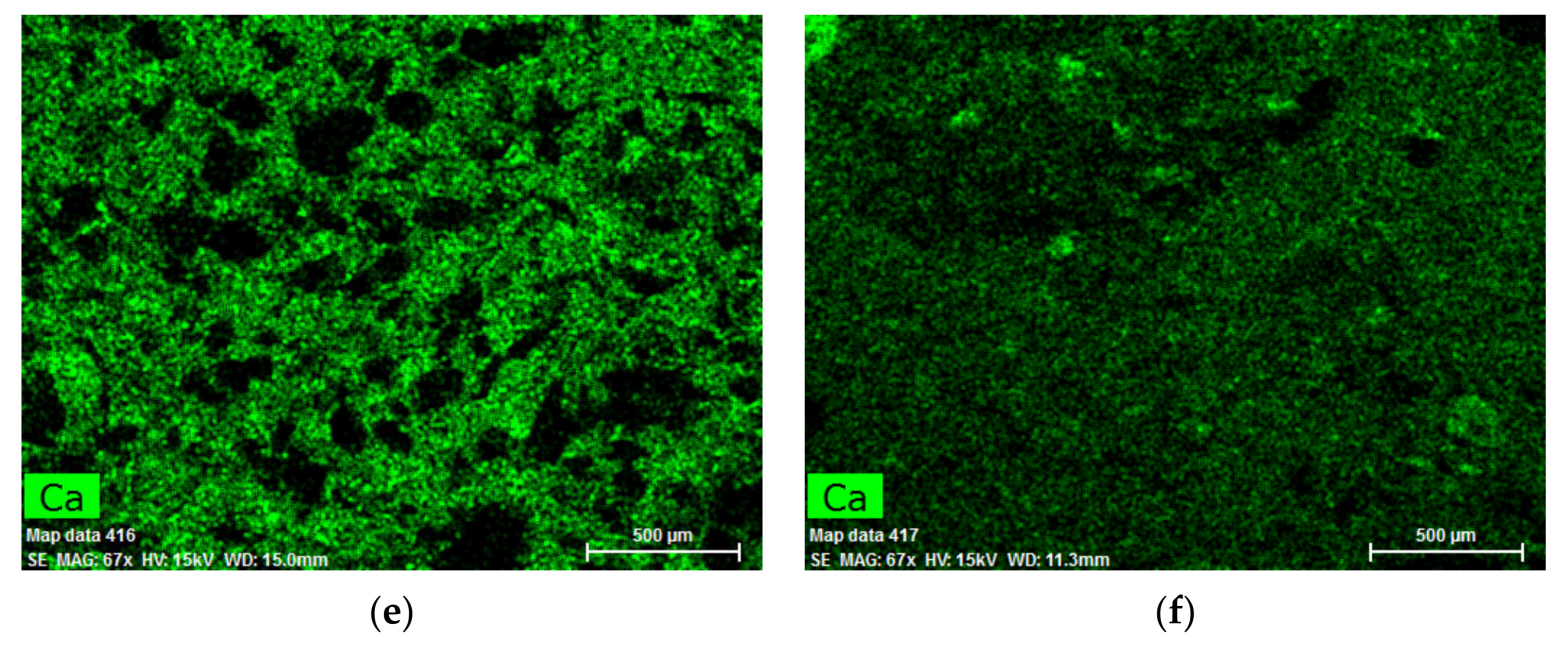
Images from the scanning electronic microscope using the EDX analysis of the cement composites with graphite filler upon 67× magnification: (**a**) SEM of the surface of the specimen with the Supragraphite C300 filler; (**b**) SEM of surface of a specimen with the Condufit C4 filler; (**c**) the red presence of the carbon in the specimen with the Supragraphite C300 filler; (**d**) the red presence of carbon in a specimen with the Condufit C4 filler; (**e**) the green presence of carbon in a specimen with the Supragraphite C300 filler; (**f**) the green presence of carbon in a specimen with the Condufit C4 filler.

The images from the scanning electron microscope using the EDX analysis (Figure 19) clearly show the differences in the distribution of the graphite filler in the matrix. While the coarse graphite is surrounded by a rather thick layer of the non-conductive hydration products and fillers, the fine graphite creates a homogenous mixture. The areas without the red-marked presence of carbon on the images are frequently depressions that could not be examined using the EDX analysis.

## 5. Discussion

The effective decrease in resistivity of silicate composites could become the key progress for modern “smart” materials that subsequently find applications in many industrial fields.

To achieve low resistivity of silicate composites while maintaining their required physical and mechanical parameters, it is imperative to understand:

- Principles of the formation of a conductive network:

Cement itself exhibits slight electron movements due to the presence of n-type conductivity. Therefore, with the addition of p-type conductive admixtures (for example, carbon particles), hole movements are present, which eventually develops electron-hole distribution in cement composites [3,97].

The results of Xu’s research show that the relative dielectric constant of the compo-sites can be increased by filling graphite powder into the cement/polymer matrix, and meanwhile the dielectric loss of the composites can also be decreased. The desired dielec-tric or electromechanical coupling properties can be tailored by filling the inorganic pow-der into the composites [101].

Using suitable types of fillers and their combinations, it is possible to create an integrated and perfectly interconnected electrically conductive structure in the composites that are capable of transmitting electrical current and is necessary for the effective decrease in resistivity.

According to the percolation theory, Xie found that in a CF/cement composite system, the critical exponent is neither a universal value nor a constant with the fillers’ concentration slightly above the percolation threshold [102].

- The effect of the structure on the resistivity of cement pastes/silicate composites:

The resistivity primarily depends on two main properties of the composite—the density and electrical conductivity of the structure. The amount and character of conductive particles directly relate to these parameters. The conductivity typically varies depending on the concentration of ions. A cementitious microstructure has continuous hydrational developments, and as a result, the porosity and connectivity of the pores vary as a time function, which improves the microstructure [25].

In the case of high density, the current is able to flashover between the individual particles using free electrons. These are closer together and there is a higher likeliness that they flashover between the valence layers of the individual atoms.

Tumidajski’s observation revealed that the electrical conductivity of the transition zone between cement paste and aggregate is not much different from the electrical conductivity of the paste. The mortar electrically conductive behavior for aggregate volume fractions greater than 0.10 can be approximated by the ideal case of insulating spheres embedded in a conductive matrix. [103].

Abdul Hamead found out that the electrical conductivity depends on the porosity and conductive particles system in the cement matrix (the good distribution of particles ensures a higher conductivity) and the microstructure’s humidity [25].

It was found that lower resistivity is achievable through a suitable composition of the aggregate, adding fine and non-conductive fillers (silica sand, limestone). This is particularly achieved through a denser structure, as well as higher amounts of functional paths for the electric current. Another factor is the use of suitable electrically conductive fillers, where the most reliable are those based on graphite. Zhang figured out that when the carbon black content is 0.1%, the conductive concrete has good conductivity and the resistivity of concrete tended to be stable around 530 Ωcm [24]. In our research, graphite-based fillers have proved to be the most effective.

- The evaluation of the character of the fillers on the resistivity of cement pastes/silicate composites with various types of fillers

**Figure 20:**
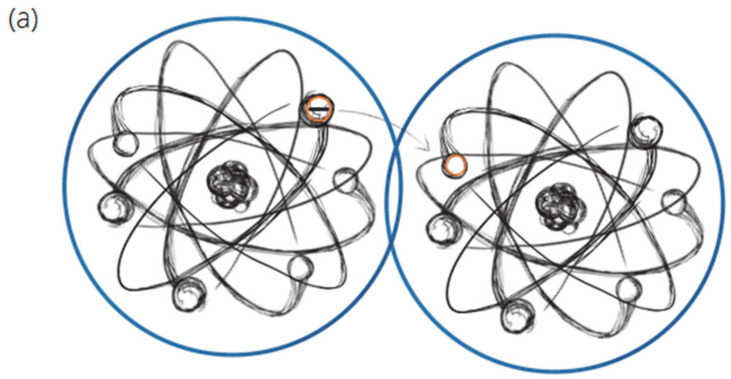

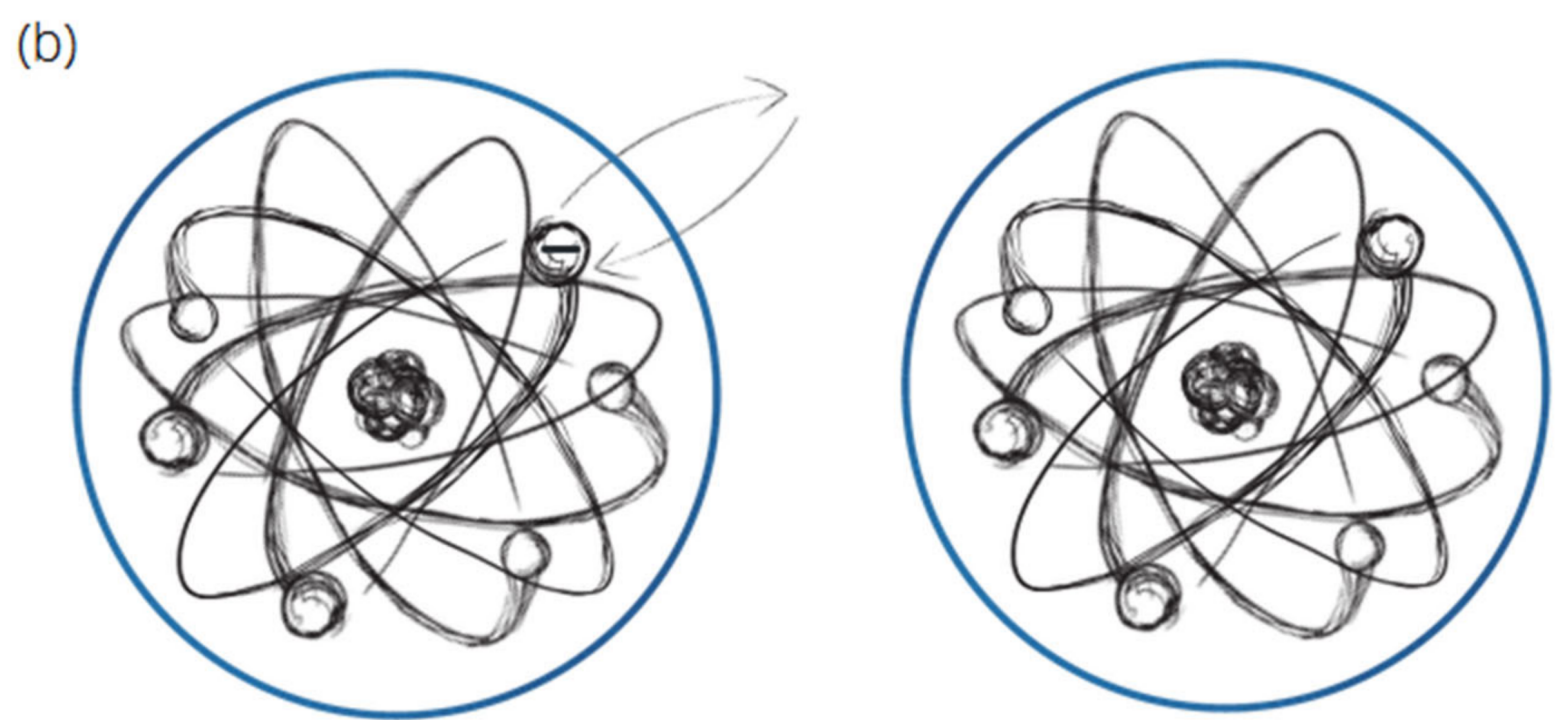
The transmission of an electrical charge in relation to the distance of the conductive elements: (**a**) conductive layers of the individual elements are intertwined, enabling transmission; (**b**) the conductive layers of the individual elements are not connected, disabling transmission.

The conductive filler creates “shortcuts” for the electric current, using low resistivity that is significantly lower (approximately a million times lower) than the resistivity of their surrounding matrix or other fillers. Lopanov [28] found out that with an increase in concentration of the conductive phase, a change of the electrical conductivity activation energy due to an increase in the number of contacts between particles is observed. In case the particles are in contact, in which the distances between them are small, it increases the likelihood of a transmission of an electrical charge. The conductivity decreases with the increasing distance, as the free movement of the electrical charge between the conductive elements is restricted (Figure 20). 

This research showed that particularly the specific surface, granularity and shape of the particles have a critical effect on the resistivity of the silicate composite while using various types of graphite. It was found that the most effective types of graphite for the formation of a conductive structure are fine types of graphite with high specific surfaces and an irregular type of particles in the shape of a “spiny sphere”. Given their shape, these particles have a higher likeliness of connecting conductive layers of the individual particles. The very fine particles of graphite help achieve higher homogenization of the material and the distribution of the conductive particles throughout the entire matrix. No significant differences were found between synthetic and natural graphite. Partial differences could be ascribed to the varied fineness of graphite. 

According to Rew’s research [75], the mastics with amorphous flake type of graphite remain non-conductive at the maximum content (25% by volume = 40% by weight). The mastic containing a synthetic graphite shows the reduced electrical resistivity, but the resistivity is higher than the mastics containing the same amount of the flake-type of graphite. Rew concludes that the scatter of the resistivity decreases as resistivity drops. The resistivity of mastics containing the flake graphite gradually decreases with the increase in the graphite content. A relatively rapid drop in resistivity exists between 13% and 16% or 10% and 13% by mastic volume, which can be considered as the percolation threshold, and then, the resistivity decreases gradually from 105 to 101 Ω·cm with increasing graphite content. Rew’s research implies that the electrical resistivity can be controlled within the range of 101–105 Ω·cm, and sufficiently high conductivity (101/Ω·cm) can be obtained only by using the flake graphite [75].

- The evaluation of the effect of water saturation on the resistivity of cement pastes/silicate composites with various types of fillers:

As is apparent from the results, the resistivity of both the pastes and composites without conductive fillers dramatically increases with the decreasing amount of water. Upon the decreasing the water contained in the specimens to under 70%, their resistivity increases up to 10×. This is caused by the reduction of free electrons provided by the electrolyte in the form of water. It was further found that in both pastes and composites with graphite fillers, the effect of humidity is dependent on the size and character of the particles of the used graphite. In the case of pastes and composites with fine types of fillers, the resistivity value decreases proportionally with decreasing amount of water in the specimen. Upon the decrease in free bound water content approximately under 70%, the resistivity is almost stable and remains so even as the reduction of the water content in the specimen continues. In the case of composites with a coarse type of filler, the resistivity increases with the reduction of humidity. The rate of resistivity increase in composites with a coarse type of filler is comparable with the growth of resistivity in the specimens without a conductive filler. This is caused by the imperfectly interconnected electrically conductive inner network that requires the presence of an electrolyte in the pore water to connect the conductive particles. Results of resistivity determination of samples with Micrographite fully correspond with Gawel’s [35] research, which comes with a finding that the presence of free water in the carbon nanofibers-cement composite pore volume contributes to the electrical connectivity loss between carbon nanofibers. Due to drying as well as during the use of no fibers (water saturated 160 Ω·cm, after drying 120 Ω·cm, i.e., approx. 25% decrease), the resistivity is reduced by the use of Micrographite, too. However, in this case, the effect of particle morphology is significant. Filler with lamellar particles with a slightly elongated shape deflected in the direction of one of the symmetry axes (Micrographite UC4) devices decrease in resistivity of cement pastes similar to nanofibers from Gawel’s study (a 22% increase in resistivity if samples were water saturated). Comparing fillers with shapes of surface-disrupted hexagonal lamellas to shapes similar to a defective circle (Micrographite C4 and Condufit C4) makes it even more noticeable that an increase in resistivity depends on the humidity (45% increase in resistivity if samples with Micrographite C4 were water saturated, and 38% by Condufit C4). Zhang’s [104] and Tzounis’s [105] hypotheses were confirmed. It presents that water molecules create an insulating layer between conductive fibers (in our research lamellar Micrographite). This layer is eliminated after drying, and at the same time, the conductivity increases. The conductivity of Gawel’s [35] sample is higher after drying because the ionic conduction in the wet state is a less efficient conduction mechanism than electronic conduction in a dry state. Our research of Micrographite confirmed this hypothesis too. However, the situation with the coarse type of graphite filler is completely different. With decreasing humidity, the resistivity rapidly increases (approximately fivefold). The dissimilarity of Zhang’s [104] and Tzounis’s [105] hypotheses was detected. It is obvious that dependence between the graphite filler size/morphology and mechanical properties, as well as the conductivity, exists.

- The evaluation of the effect of type of fillers on the resistivity of cement pastes and silicate composites:

The interfacial region, whose porosity and electrical conductivity exceed those of the bulk paste, percolate at high solid contents. A percolating pathway appeared for aggregate volume fractions higher than 60% [106]. This research examined the effects of the character of graphite-based fillers on the resistivity of cement pastes and silicate composites. It became apparent that the actual cement paste with graphite shows lower resistivity in the case of specimens saturated with water than in the case of water-saturated specimens of the composites. The decreasing ratio of water in the specimens with a coarse type of graphite then significantly increased resistivity of the composite in comparison to the cement paste. In the case of fine types of graphite, the decreasing ratio of water in the specimen decreased resistivity in both the pastes and composites. In the case of dried specimens with fine graphite, 2× lower values of resistivity were reached than in the case of cement-paste specimens. Cement itself is well known for its ionic conductivity, which is particularly high when the cement contains a substantial amount of free water. However, the thermoelectric behavior of carbon particles reinforced cement indicates electronic conductivity (provided by hole conductivity) as the main carrier [106,107,108,109,110]. This is caused by the content of silica sands as fillers that, unlike the cement paste, have lower resistivity. This is the result of an incomplete filling of the valence layers that enable conductivity in silica, while the formed C-S-H gels in the cement paste are, from the perspective of electric conductivity, contain more non-conductors.

- The evaluation of the effects of the character of the filler on the strength of cement pastes/silicate composites with various types of fillers:

Different from the repulsion energy of similar particles, heterogeneous particles pre-sent an electrostatic attraction interaction when the electric potential difference between particles is great, even though they exhibit similar surface charges [111]. Strong agglomeration of graphite particles can be observed, which can be attributed to their great total attractive energy [111].

Our research showed that the use of the coarse type of graphite in the cement paste has shown a positive effect of increasing the flexural tensile strength by 20%. Upon using the fine types of fillers, strength decreased by approximately 30% in all three types. In the case of the composites, in all types of graphite, there were reductions in the flexural tensile strength. Specifically, it was a 34% decrease in the case of the coarse type of graphite and an 82% decrease in the case of fine types of graphite. 

Compression strength dramatically decreases with the addition of graphite fillers into the cement paste. Upon using the coarse type of graphite, the decrease was 67%, and upon using fine types of graphite, all three had a comparable decrease of approximately 78%. In the case of composites, the effect of fine graphite on the compression strength was very apparent, reducing the strength by approximately 90%. The addition of a coarse type of graphite resulted in a smaller decrease of approximately 58%. Some authors [112,113] noticed better mechanical properties, mainly better workability caused by the larger lateral size of graphite grains—the lubricating effect overcame the thickening action. Better workability caused higher density, lower pore content and better sample compactness. Better structure led to higher flexural and compressive strength. Lamastra [114] observed the impact of self-lubricating particles on flowability only at very low graphite nano pellets content. A decrease in the flowability parameters at a higher dosage of filler was observed, and this effect was assigned to mortar granulometry and lateral particle size of graphite grains similar to in our research. The strength decrease similar to our achieved results was described by Plugin [26] in his study, too. He described that the graphite does not participate in the hydration of Portland cement and scarcely affects the composition of its hydration products, except for a certain decrease in the degree of hydration of cement and the basicity of calcium hydrosilicates as in [26]. 

## 6. Conclusions

It may be concluded that graphite materials featuring very fine particles and high specific surface are most suitable and most effective for electrically conductive silicate composites. From the perspective of particle shapes, the most suitable round particles with a coarse surface can be achieved, for example, through the use of nanoparticles, such as the Condufit C4 type of graphite. The amount, shape and, in particular, the fineness of the graphite filler particles thus creates suitable conditions for the creation of an integrated internal electrically conductive network. In the case of the use of a coarse type of graphite or purely non-conductive fillers, the presence of an electrolyte, for example, in the form of water, is necessary to achieve a low resistivity. The specific conclusions of the presented work are as follows:

- Principles of the formation of a conductive network:

It has been confirmed that by using suitable types of fillers, especially graphite-based fillers, it is possible to create an integrated and perfectly interconnected electrically conductive structure in the composites that are capable of transmitting an electrical current and is necessary for the effective decrease in resistivity.

- The effect of the structure on the resistivity of cement pastes/silicate composites:

It was found that lower resistivity is achievable not only through electrically conductive fillers but also by achieving a denser structure of the composite and a suitable composition of the aggregate and adding fine and non-conductive fillers (silica sand, limestone).

- The evaluation of the character of the fillers on the resistivity of cement pastes/silicate composites with various types of fillers:

It was found that the most effective types of graphite for the formation of a conductive structure are fine types of graphite with high specific surfaces and irregular types of particles. These particles have a higher likeliness of connecting conductive layers of the individual particles. The very fine particles of graphite help achieve higher homogeneity of the material. No significant differences were found between synthetic and natural graphite.

- The evaluation of the effect of water saturation on the resistivity of cement pastes/silicate composites with various types of fillers:

As is apparent from the results, the resistivity of samples without conductive fillers increases up to 10× with the decreasing amount of water. In the case of samples with fine types of graphite fillers, the resistivity value is almost stable and remains so even with a decreased amount of water in the specimen. In the case of composites with a coarse type of graphite filler, the resistivity increases with the reduction of humidity.

- The evaluation of the effect of type of fillers on the resistivity of cement pastes and silicate composites:

It became apparent that the water-saturated cement paste with graphite shows lower resistivity than water-saturated cement composites. The decreasing ratio of water in the specimens with a coarse type of graphite significantly increased the resistivity of the composite in comparison with the cement paste. In the case of fine types of graphite, the decreasing ratio of water in the specimen decreased resistivity in both the pastes and composites.

- The evaluation of the effects of the character of the filler on the strength of cement pastes/silicate composites with various types of fillers:

Compression strength dramatically decreases with the addition of graphite fillers in both the pastes and composites. Upon using the coarse type of graphite, the decrease was 62% on average and upon using fine types of graphite, 84% on average.

## Figures and Tables

**Table 1:** Properties of the CEM I 42,5 R cement.

Selected Properties	CEM I 42,5 R
Specific surface (m^2^/kg)	391 (EN 196-6, Blaine)
Volumetric mass density (kg/m^3^)	3 110
Resistivity (Ω·m)	9.12 × 10^6^

**Table 2:** Selected properties of sands.

Selected Properties	Silica Sand 1.6/4	Silica Sand 30/31	Silica Sand 35	Lime Stone VBS 40
Bulk density loose (kg/m^3^)	1540	1460	1380	1050
Bulk density of compacted aggregate (kg/m^3^)	1590	1600	1470	1190
Water absorption capacity WA5 (%)	3	4	11	22
Volumetric mass density (pycnometric) (kg/m^3^)	3120	2630	3380	2660
Resistivity (Ω·m)	*	*	*	38.89 × 10^6^
Grain size (mm)	1.4–4.0	0.3–1.0	0.1–0.3	0.0–0.4

*—immeasurable.

**Table 3:** Selected properties of the plasticizer STACHEMENT 2180.1.

Selected Properties	Plasticizer
Density (kg/m^3^)	1055 ± 20
Dry mass content (% of weight)	25 ± 2
Maximum chloride content (% of weight)	0.1
Maximum alkaline content (% equivalent to Na_2_O)	2
Recommended amount (% of the cement weight)	0.4–1.4

**Table 4:** Classification of primary fillers.

Commercial Name	Granularity	Genesis
Supragraphite C300	Coarse	Natural
Micrographite UC4	Fine	Synthetic
Micrographite C4	Fine	Natural
Condufit C4 *	Fine	Natural

*—graphite with improved electric conductivity properties.

**Table 5:** Selected properties of used graphite types.

Selected Properties	Unit	Supragraphite C 300	Micrographite UC 4	Micrographite C4	Condufit C4
Particle type		Flat flakes	Flat, irregular	Irregular	Irregular with rough surface
Bulk density loose	(kg/m^3^)	450	170	150	180
Bulk density of compacted aggregate	(kg/m^3^)	550	220	190	230
Volumetric mass density (pycnometric)	(kg/m^3^)	2200	2060	2100	2100
Volumetric weight (helium pycnometer)	(kg/m^3^)	2250	2650	2500	2410
Granularity D (0.1)	(μm)	83.118	1.994	2.004	1.504
D (0.5)	(μm)	197.019	3.478	3.375	2.905
D (0.9)	(μm)	390.432	5.884	5.487	5.261
Specific surface	(m^2^/kg)	1194	12454	11933	20418
Water absorption capacity WA (5)	(%)	26	174	172	190
Resistivity	(Ω·m)	1.06	1.33	1.95	1.68

**Table 6:** Comparison of properties of used materials.

		Resistivity (Ω·m)	Water Absorption Capacity (WA5 %)	Particle Size (mm)	Volumetric Weight (kg/m^3^)
Basic Materials	Cement CEM I 42,5 R	9.12 × 10^6^	*	0.0–0.25	3100
	Limestone VBS 40	38.89 × 10^6^	22	0.0–0.4	2660
	Silica sand 30/31	*	4	0.3–1.0	2630
	Silica sand 35	*	11	0.1–0.3	3380
	Silica sand 1,6-4	*	3	1.4–4.0	3120
Conductive Fillers (Graphite powder)	Supragraphite C300	1.06	26	0.10–0.25	2200
	Micrographite UC4	1.33	174	3.5–5.0 × 10^3^	2060
	Micrographite C4	1.95	172	3.5–5.0 × 10^3^	2100
	Condufit C4	1.68	190	3.5–5.0 × 10^3^	2110

*—immeasurable.

**Table 7:** The composition of cement pastes with the addition of conductive fillers.

Type of Admixture	Amount (Weight in %)		w/c Ratio
	CEM I 42,5 R	Graphite	
Reference mixture	100%	0%	0.35
Supragraphite C 300	80%	20%	0.48
Micrographite UC 4	80%	20%	0.65
Micrographite C 4	80%	20%	0.65
Condufit C4	80%	20%	0.72

**Table 8:** Composition of the mixture for the verification of the resistivity of the composite.

Component	Amount (Weight in %)	
	Reference Mixture	Mixture with the Conductive Filler
CEM I 42,5 R Portland Cement	21.0%	16.8%
VBS40 Micro-ground Limestone	8.4%	6.7%
Mixture of Silica Sands 0–4 mm	70.5%	56.4%
Plasticizer	0.2%	0.1%
Conductive Filler	0.00%	20.0%

## Data Availability

Data is contained within the article material.
